# Nanocomposites comprised of homogeneously dispersed magnetic iron-oxide nanoparticles and poly(methyl methacrylate)

**DOI:** 10.3762/bjnano.9.153

**Published:** 2018-06-01

**Authors:** Sašo Gyergyek, David Pahovnik, Ema Žagar, Alenka Mertelj, Rok Kostanjšek, Miloš Beković, Marko Jagodič, Heinrich Hofmann, Darko Makovec

**Affiliations:** 1 Department for Materials Synthesis, Jožef Stefan Institute, Jamova 39, 1000 Ljubljana, Slovenia; 2 Faculty of Chemistry and Chemical Engineering, University of Maribor, Smetanova 17, 2000 Maribor, Slovenia; 3 Department of Polymer Chemistry and Technology, National Institute of Chemistry, Hajdrihova 19, 1000 Ljubljana, Slovenia; 4 Complex Matter, Jožef Stefan Institute, Jamova 39, 1000 Ljubljana, Slovenia; 5 Department of Biology, Biotechnical Faculty, University of Ljubljana, Večna pot 111, 1000 Ljubljana, Slovenia; 6 Institute of Electrical Power Engineering, Faculty of Electrical Engineering and Computer Science, University of Maribor, Smetanova 17, 2000 Maribor, Slovenia; 7 Institute of Mathematics, Physics and Mechanics, Jadranska 19, 1000 Ljubljana, Slovenia,; 8 Laboratory for Powder Technology, Ecole Polytechniquie Fédérale de Lausane, Station 12, 1015 Lausane, Switzerland

**Keywords:** magnetic hyperthermia, magnetic properties, nanocomposites, superparamagnetic

## Abstract

Nanocomposites with a high, uniform loading of magnetic nanoparticles are very desirable for applications such as electromagnetic shielding and cancer treatment based on magnetically induced hyperthermia. In this study, a simple and scalable route for preparing nanocomposites with a high, uniform loading of magnetic nanoparticles is presented. The magnetic iron-oxide nanoparticles were functionalized with a methacrylate-based monomer that copolymerized in a toluene solution with the methyl methacrylate (MMA) monomer. The resulting suspension of magnetic nanoparticles decorated with poly(methyl methacrylate) (PMMA) chains in toluene were colloidal, even in the presence of a magnetic field gradient. Nanocomposites were precipitated from these suspensions. The transmission electron microscopy investigation of the prepared nanocomposites revealed that the magnetic nanoparticles were homogeneously dispersed in the PMMA matrix, even in amounts up to 53 wt %. The uniform dispersion of the nanoparticles in the PMMA matrix was attributed to the good solvation of the grafted PMMA chains from the magnetic nanoparticles by the PMMA chains of the matrix. The nanocomposites were superparamagnetic and exhibited large values for the saturation magnetization of up to 36 emu/g. Moreover, the nanocomposite with the largest amount of incorporated nanoparticles exhibited relatively large values for the specific power loss when subjected to alternating magnetic fields, giving this material great potential for the magnetically induced hyperthermia-based treatment of cancer.

## Introduction

Magnetic iron-oxide nanoparticles with a size close to the superparamagnetic limit have been extensively studied because of their unique properties that can be exploited in a variety of applications. When the size of a single-domain ferromagnetic material is reduced below a certain critical value, the transition to the superparamagnetic state is observed. Above a certain critical temperature, called the blocking temperature, the thermal energy induces rapid fluctuations of the nanoparticle’s magnetic moment relative to the time of observation [1–2]. Superparamagnetic nanoparticles do not show remanence and coercivity in the absence of an external magnetic field [1–2]. Their colloidal suspensions are vital in a variety of technological [3] and biomedical applications [4], such as contrast agents in magnetic resonance imaging (MRI) [5–6], targeted drug delivery [6] and magnetic hyperthermia based on the selective heating of magnetic nanoparticles using an external AC magnetic field [7–8].

While magnetic nanoparticles exhibit unique physico-chemical properties, they also tend to agglomerate, leading to the loss of their interesting properties and their potential for applications. Organic/inorganic nanocomposites combine the unique properties of nanoparticles with the advantages of a polymer matrix, which include low weight and easy formability [9–12]. Injectable formulations that gel in the targeted tumor and simultaneously entrap magnetic nanoparticles in the polymer were successfully used to deliver local magnetic hyperthermia [13]. One of the key properties of the formulation was the colloidal stability of the magnetic nanoparticles in the polymer solution. Acrylic-based cements are extensively used for the fixation of implantable prosthesis or in vertebroplasty, i.e., the procedure to fix fractured vertebra. Vertebra tumors fracture vertebra and could be treated by magnetically induced hyperthermia with the same material as used for vertebroplasty if magnetic nanoparticles were incorporated. Acrylic-based bone cements are based on mixing a liquid component with a powder to form a dough-like putty that hardens at body temperature [14]. Typically, the liquid component is (MMA) and the powder component is a polymer with additives that enable rapid hardening at body temperature [14]. One of such polymers is poly(methyl methacrylate) (PMMA). It is an amorphous, linear thermoplastic having good mechanical properties, exceptional optical clarity as well as being biologically inert [15–16]. In order to preserve the unique properties of the nanoparticles they should be homogeneously dispersed within the polymer matrix. The surface of the magnetic nanoparticles is generally hydrophilic and thus not compatible with hydrophobic polymers, such as PMMA. Usually, oleic or ricinoleic acid (RA) is bound to the surface of the nanoparticles to make them hydrophobic [17–18]. However, this is usually insufficient to prevent depletion flocculation in the polymer solution or polymer melt [19–21]. This problem was successfully overcome by using polymer-grafted nanoparticles (magnetic and silica), i.e., composite nanoparticles that were mixed with the polymer, usually in the solution [10,22–24]. Critical factors that influence the state of dispersion of polymer-grafted nanoparticles in nanocomposites are grafting density, molar mass of grafted chains (brushes) and conformation of brushes. Grafting density strongly influences chain conformation. At a large grafting density, brushes cannot adopt a random coil conformation but instead adopt a stretched brush conformation and their length (*h*) is larger than radius of gyration (*R*_g_) of the random coil of comparable molar mass. Xu et al., for example, obtained nanocomposites with well-dispersed polymer-grafted nanoparticles when the brush thickness, *h*, exceeded 7.5 nm and, *h*/*R*_g_, was larger than 1.8, even in the case when the molar mass of the matrix polymer was almost twice as large as that of the brush [23]. Moll et al. obtained a similar state of dispersion only when the molar mass of the brush was almost four times larger than that of the matrix when using a significantly lower grafting density than Xu [25]. Clearly, sufficiently long polymer chains that are attached to the surface of the nanoparticles that are well-wetted with the matrix polymer chains are needed for colloidal stabilization [23–26].

Colloidal suspensions of nanoparticles dispersed in a polymer solution have some advantages over bulk nanocomposites. They enable the preparation of nanocomposite films by spin coating and the precipitation of a nanocomposite in a form that is easily milled into powders, for example. Therefore, it is desirable to develop synthesis methods to prepare them. In this paper we present a simple method for preparing magnetic iron oxide/PMMA nanocomposites with a high loading of homogeneously dispersed nanoparticles. The methacrylate-monomer-functionalized magnetic iron-oxide nanoparticles were copolymerized with the MMA monomer in a colloidal suspension. The developed copolymerization procedure has two benefits: firstly, the magnetic nanoparticles are decorated with the PMMA chains that provide colloidal stability in the polymer solution, and secondly, the PMMA matrix is formed simultaneously in a one-pot synthesis. The nanocomposites were precipitated from the colloidal suspension and comprehensively characterized. In the next step, the magnetic properties of the prepared nanocomposites were studied, and their potential for hyperthermia treatment was evaluated.

## Experimental

### Synthesis

Ricinoleic-acid-coated iron-oxide nanoparticles (NP-RA) were synthesized using the hydrothermal method at 180 °C and with a molar ratio of *m*_RA_/*m*_p_ = 0.53 [27]. The synthesized nanoparticles were dispersed in toluene and the suspension was centrifuged at a relative centrifugal force (RCF) of 5000*g* for 5 min. The colloidal suspension was decanted from the centrifuge tube and analyzed using thermogravimetric analysis (TGA). The colloidal suspension was composed of 37.8 wt % nanoparticles (NPs), 4.1 wt % ricinoleic acid and 58.1 wt % toluene.

**Scheme 1:**
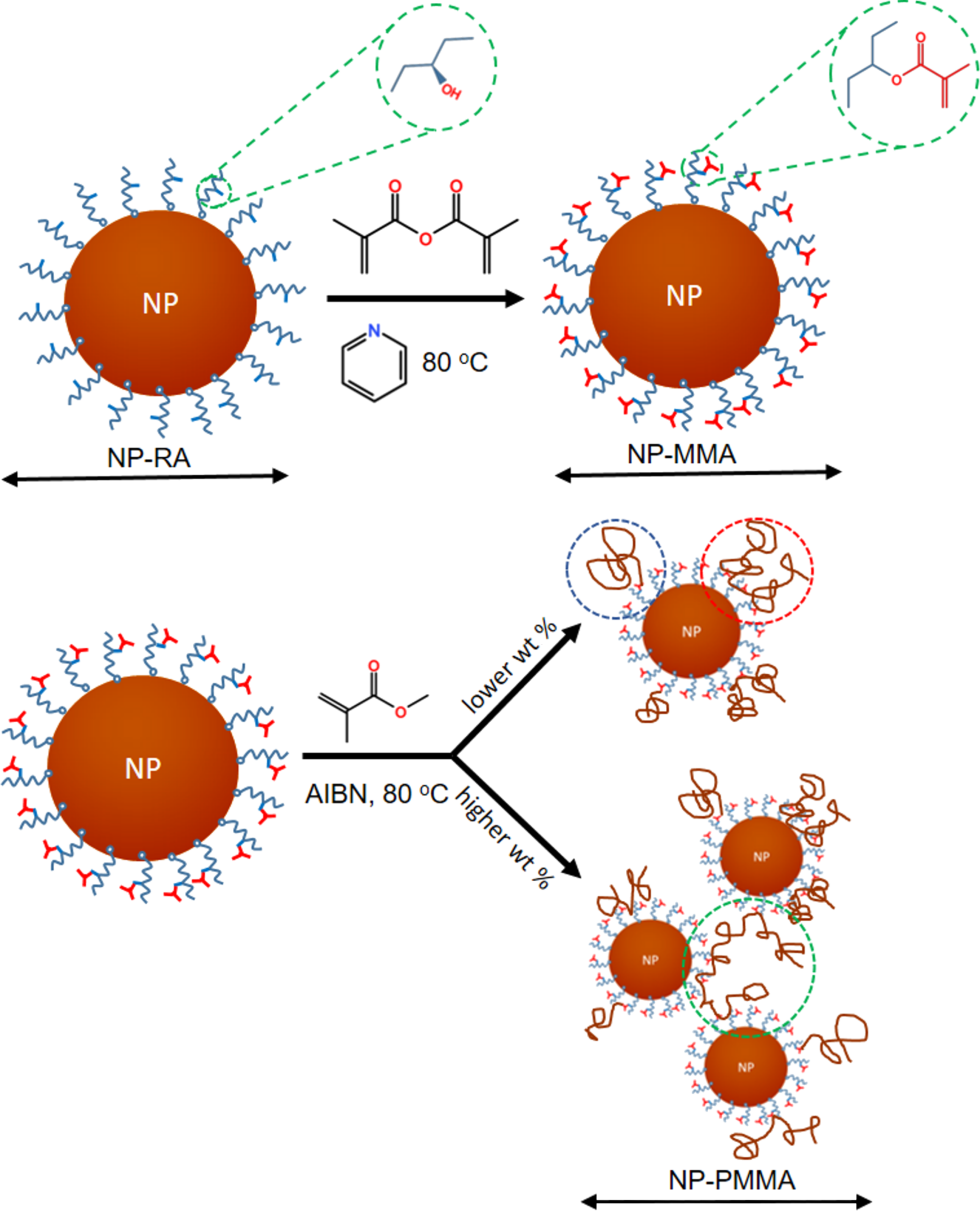
Illustration of the possible copolymerization reactions involved in the preparation of the nanocomposites.

The grafting of the methacrylic units to the nanoparticle surface (NP-MMA) was achieved by esterification of the hydroxyl group from the ricinoleic acid with methacrylic anhydride (Scheme 1). A round-bottom flask was charged with 25 g of the suspension of NP-RA, 218 μL of methacrylic anhydride (1.37 mmol) and 110 μL (1.37 mmol) of pyridine. The flask was sealed with a septum and heated in an oil bath at 80 °C for 48 h. After cooling to room temperature, the suspension was centrifuged at an RCF of 5000*g* for 5 min. The colloidal suspension was decanted from the centrifuge tube and analyzed using TGA. The colloidal suspension contained 38.0 wt % nanoparticles (NPs). For further analyses, a small amount of the functionalized NP-MMA was isolated from the suspension by flocculating them with a large amount of acetone. The NP-MMA material was thoroughly washed with acetone and dried in an oven at 60 °C.

The nanocomposites were prepared by solution copolymerization of the MMA and NP-MMA in toluene (Scheme 1). A 100 mL three-neck round-bottom flask was charged with the suspension of NP-MMA, azobisisobutyronitrile (AIBN) (0.1 g) and bubbled with Ar. After 30 min, the bubbling was stopped, and 11 mL of MMA was added and the suspension was heated at 80 °C for 4 h. The nanocomposite (NC) was precipitated from the cooled suspension of NP-PMMA with methanol (1:10 v/v). The NC was then washed several times with methanol, oven dried at 60 °C and crushed in an agate mortar. The suspensions NP-PMMA-1, NP-PMMA-2 and NP-PMMA-3 contained 1.2 wt %, 8.1 wt %, and 26.6 wt % of the NPs, respectively. The nanocomposites NC-1, NC-2, and NC-3 were prepared from the NP-PMMA suspensions denominated with the same number. For the analysis, a small amount of the nanoparticles were isolated from the suspension of NP-PMMA-3 immediately after the polymerization by centrifugation at 20,000*g* for 20 min. Any free polymer was washed from the sample by dispersing the sediment in acetone, centrifugation at 20,000*g* for 20 min and discarding the supernatant. The dispersion/centrifugation cycle was repeated four times. The isolated NP-PMMA-3 sample was oven dried at 60 °C. Pure PMMA (sPMMA) was prepared by polymerization of the MMA under identical conditions. The bonding of the MMA to RA (RA-MMA) and copolymerization of the RA-MMA with MMA was conducted under similar conditions (see Supporting Information File 1).

A nanocomposite prepared without bonding the MMA to the surface of the nanoparticles (NP-RA-PMMA) was prepared by mixing the NP-RA suspension with the sPMMA solution in toluene (see Supporting Information File 1).

The dissolution of the Fe cations from the nanocomposite and the nanoparticles was tested with dissolution in the presence of citric acid at pH 1, where further details can be found in Supporting Information File 1.

### Characterization

The particle size, crystallinity and dispersion over the nanocomposites were characterized by transmission electron microscopy (TEM). ^1^H NMR spectra were recorded in CDCl_3_ on a 300 MHz Agilent Technologies DD2 NMR spectrometer in the pulse Fourier transform mode with both a relaxation delay and an acquisition time of 5 s. Tetramethylsilane (TMS, δ = 0) was used as an internal chemical-shift standard. The diffuse reflectance infrared Fourier transform spectra (DRIFT, Perkin Elmer Spectrum 400 equipped with DRIFT accessory) were recorded in KBr. The weight fraction of nanoparticles in the nanocomposites was determined with a Mettler Toledo thermogravimetric analysis (TGA) instrument equipped with STARe 9.3 software (Mettler Toledo, OH, USA). The glass-transition temperature of the nanocomposites was determined on a Mettler Toledo DSC1 instrument. Dynamic light scattering (DLS) was used to measure the distribution of the hydrodynamic diameter of the nanoparticle. The room temperature magnetization curves of the nanoparticles were measured with a Lake Shore 7307 vibrating-sample magnetometer (VSM). The temperature dependency of the magnetic susceptibility under zero-field-cooling (ZFC) conditions was measured with a Quantum Design MPMS superconducting quantum interference device (SQUID). The specific power loss of the nanoparticles in powder form was measured using a custom-built device [28]. See the Supporting Information File 1 for more details.

## Results and Discussion

**Figure 1:**
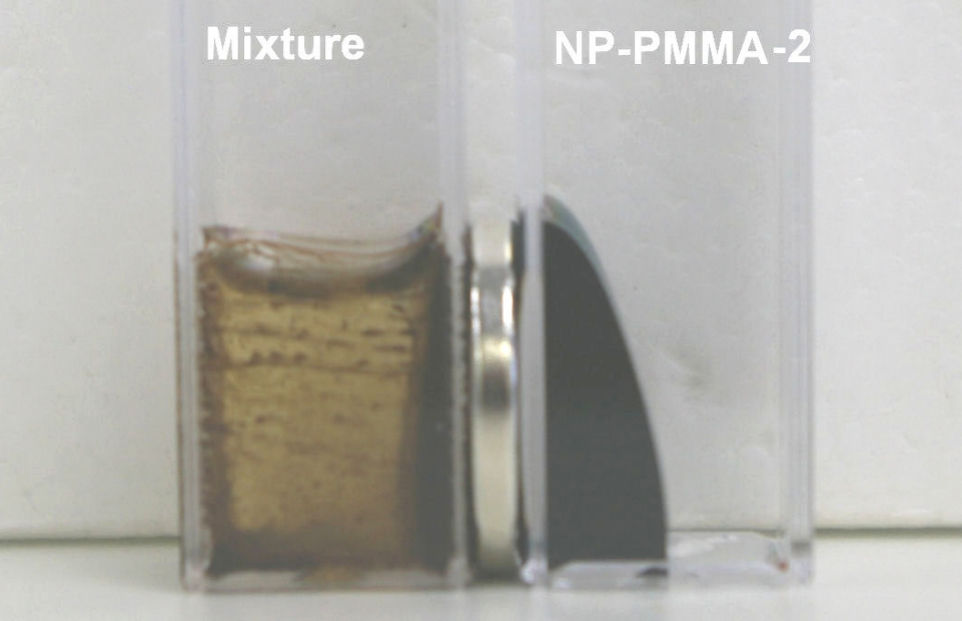
Mixture of ricinoleic-acid-coated iron-oxide nanoparticles with pure PMMA in toluene, NP-RA-PMMA (left container), and a colloidal suspension of the NP-PMMA-2 after polymerization (right container). The silver-grey disk between the containers is a permanent magnet.

Mixing the colloidal suspension of hydrophobic RA-coated iron-oxide nanoparticles, NP-RA, in toluene with a solution of sPMMA in toluene resulted in the rapid flocculation of the iron-oxide nanoparticles, which was macroscopically visible as a phase separation (Figure 1).

This phase separation was more pronounced when a small permanent magnet was placed beside the container, as shown in Figure 1. The flocculation is most probably the result of the depletion layer surrounding the nanoparticles, causing their flocculation to decrease the excluded volume of polymer chains [19–21,29]. To test if any of the added compounds stabilize the suspension of NPs in the PMMA polymer solution, the polymerization of MMA was conducted in the suspension of NP-RA in toluene to which methacrylic anhydride and pyridine were added. At the beginning of the polymerization the suspension was colloidal; however, after a short time of polymerization, the macroscopic phase separation was clearly visible. In contrast, the suspension of NP-MMA was colloidal when mixed with the MMA as well as during the MMA polymerization. The NP-PMMA-1 and NP-PMMA-2 suspensions remained colloidal for an extended time (more than a year), even in the presence of a relatively strong magnetic field gradient (Figure 1). The NP-PMMA-3 suspension was a viscous, free flowing, swollen gel that after a prolonged time (≈24 h) self-separated into a colloidal suspension and a gel.

**Figure 2:**
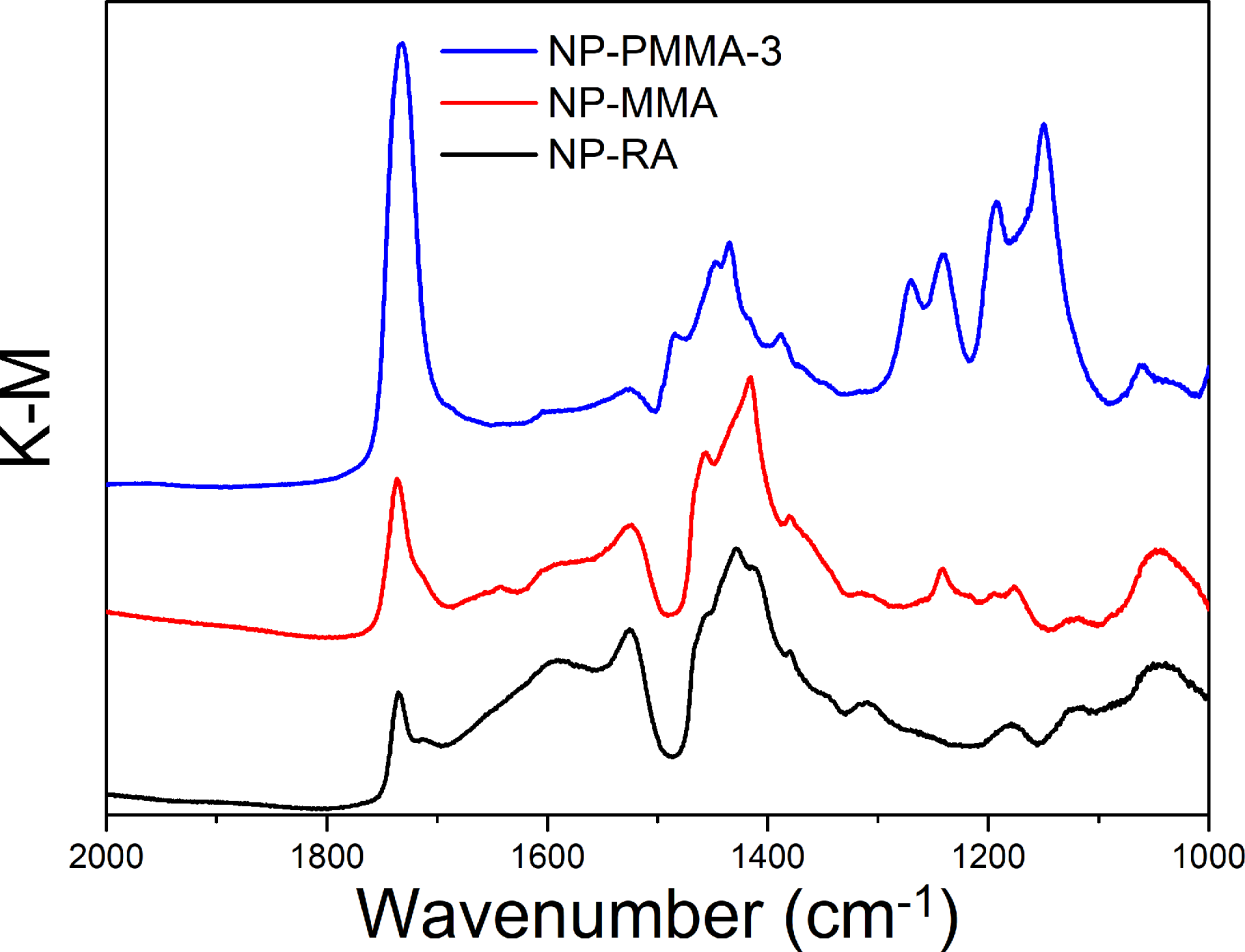
DRIFT spectra of the nanoparticle samples NP-RA, NP-MMA and NP-PMMA-3.

Recent experimental and theoretical work on depletion forces has shown that depletion attraction is actually the short-range component of a more general structural interaction [19]. When higher-order concentration effects are taken into account, long-range oscillatory interaction energy profiles were measured and predicted, meaning that the so-called depletion stabilization can occur when a simple treatment would suggest flocculation [19]. The details of the profile are mostly influenced by the concentration of macromolecules and the second-order virial coefficient. The colloidal stability of NP-PMMA in the polymer solution is clearly related to the presence of the polymer chains that are strongly bonded to the surface of the NP-PMMA. The origin of the stabilization is beyond of the scope of this paper. RA has a hydroxyl group in the chain, which can be esterified using methacryloyl chloride or methacrylic anhydride to prepare RA-based methacrylate monomers. This possibility was checked with the RA in the form it was received from the supplier under reaction conditions that were used with the nanoparticles. It is worth mentioning that a part of the hydroxyl groups in the original RA of technical grade (80% purity) was already esterified, as indicated by a small intensity peak at 4.88 ppm and a shoulder on the left-hand side of the vibration band of the carbonyl group in its ^1^H NMR and FTIR spectra, respectively (Supporting Information File 1, Figures S1 and S2). The shift of the peak for the methine group, denoted with D, from 3.62 ppm in the ^1^H NMR spectrum of the original ricinoleic acid to 4.88 ppm in the ^1^H NMR spectrum of RA-MMA, and a pronounced increase in intensity of the left-hand side of the vibration band of the carbonyl group confirm the formation of the methacrylic ester of RA (Supporting Information File 1, Figures S3, S4 and S5). The disappearance of the peak representing the MMA double bond in the ^1^H NMR spectrum of the RA-PMMA reveals the successful copolymerization of the RA-MMA with the neat MMA (Supporting Information File 1, Figure S4). The DRIFT spectrum of the NP-RA showed absorption peaks at 1520 cm^−1^ (ν_a_ COO^−^) and 1417 cm^−1^ (ν_s_ COO^−^) (Figure 2). The difference Δν = ν_a_(COO^−^) − ν_s_(COO^−^) is 103 cm^−1^. According to Deacon and Philips [30], the change in the carbon–oxygen stretching vibration and the difference between the asymmetric and symmetric stretching-vibration frequencies relative to the free carboxylate ion are indicative of a mononuclear bidentate complex. The absorption peak at 1736 cm^−1^, indicative of ester groups, is significantly enhanced in the NP-MMA sample when compared to the NP-RA sample (Figure 2), indicating the formation of the methacrylate ester, as depicted in Scheme 1. After polymerization (NP-PMMA-3) the FTIR spectrum shows the characteristic features of PMMA (Figure 2). Based on these results we can conclude that the hydroxyl group of the ricinoleic acid bonded to the surface of the magnetic nanoparticles was esterified with methacrylate, leading to the copolymerization of NP-MMA and MMA. The polymerization can be considered as the reaction of vinyl monomers and multi-vinyl monomers for the preparation of hyperbranched polymers [31].

Three different events can lead to the copolymerization and attachment of the PMMA chains to NP-MMA: i) initiation occurs on the NP-MMA, resulting in PMMA chain(s) growing from the surface of the NP-MMA (blue circle in Scheme 1); ii) growing PMMA chain(s) copolymerizes with the methacrylate unit(s) on the surface of a single NP-MMA (red circle in Scheme 1); iii) growing PMMA chain copolymerizes with methacrylate unit(s) on the surface of more than one NP-MMA particle, resulting in the formation of covalent links between the NP-MMA surfaces (green circle in Scheme 1).

Of the three events, the last one is the most dependent on the concentration of NP-MMA and can even lead to cross-linking. The relative occurrence of this event should have a major influence on the colloidal stability of the NP-PMMA suspension and the hydrodynamic size of the NP-PMMA. The gel properties of the suspension of the NP-PMMA-3, where the concentration of the NP-MMA was the highest, indicate the relatively high content of the PMMA chains connecting several neighboring nanoparticles.

**Figure 3:**
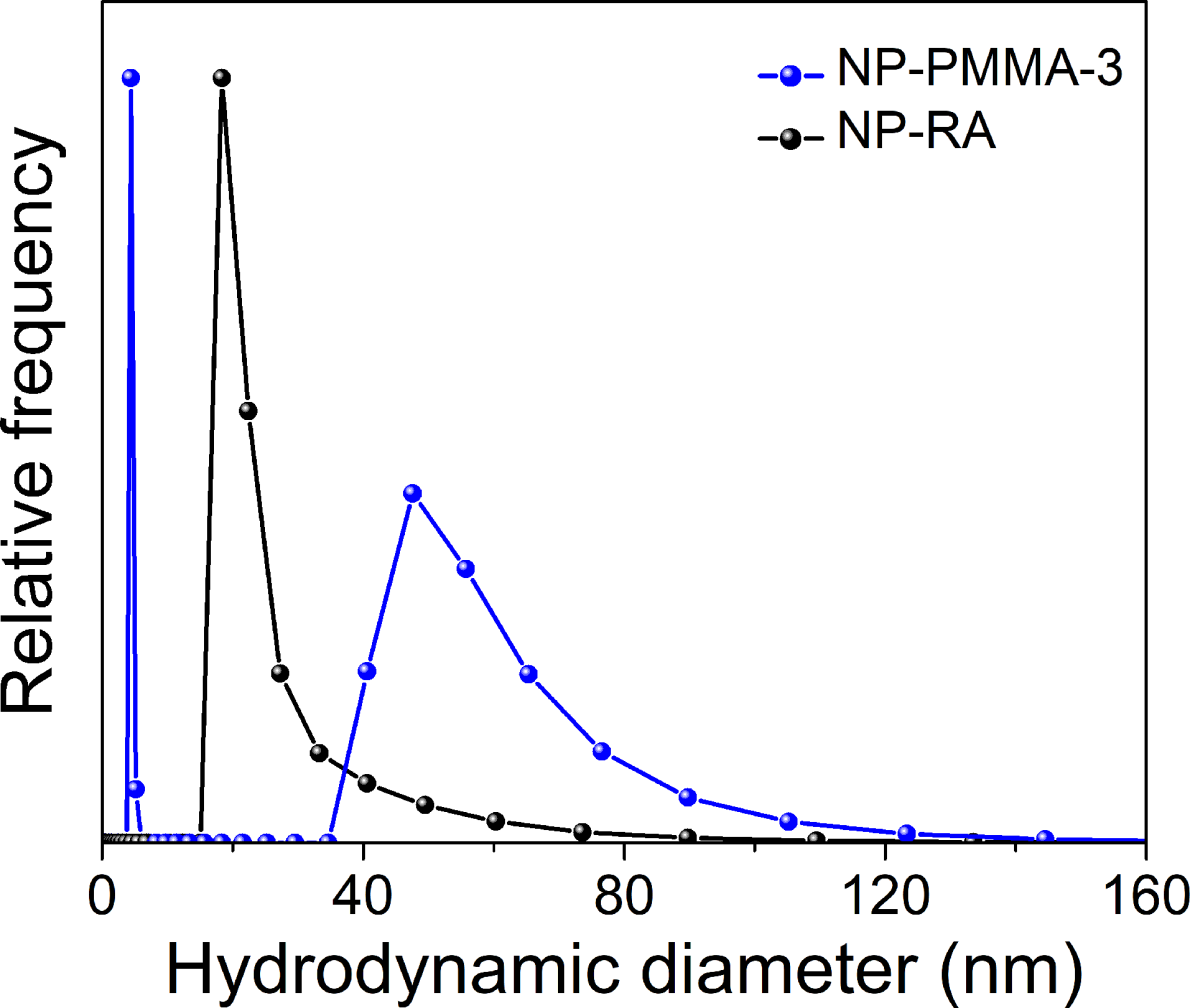
Number-weighted hydrodynamic diameter size-distribution function of the NP-RA and NP-PMMA-3 nanoparticle samples.

The size of the NP-PMMA-3 nanoparticles and the free PMMA chains were probed using dynamic light scattering (DLS). Two distinct populations were found in the suspension of nanoparticles denoted as NP-PMMA-3 (Figure 3).

The distribution function for the smaller diameter region was extremely narrow, with the mode at 2 nm. The distribution function for the larger diameter region is strongly skewed and the median, having the value of 45 nm, is a better estimate of the “average” hydrodynamic diameter than the mean or mode. The distribution functions were well separated; therefore, we can conclude that the one centered at 2 nm described the hydrodynamic size of the free polymer chains, and the other one, the NP-PMMA nanoparticles. Additionally, the sPMMA sample was measured. In this case the signal was weak so the autocorrelation function was acquired at a low angle, i.e., at a small scattering vector that makes it possible to probe the fast dynamics of small polymer chains. The resulting distribution function of the hydrodynamic size of the polymer chains was centered at approximately 4 ± 2 nm. The large error is due to the weak signal. On the other hand, in the NP-PMMA-3 sample, the scattering from the nanoparticles overwhelmed the scattering from the small polymer chains, also making an estimation of their size less reliable. We believe that the hydrodynamic diameter of the free PMMA chains being between 1 nm and 4 nm is a valid assumption. The distribution function of the NP-RA nanoparticles is less skewed with a median of 30 nm. The number-weighted average size and the median of the nanoparticles NP-RA determined from the TEM images were 11.0 ± 2.3 nm and 10 nm, respectively (Supporting Information File 1, Figure S6). Considering the fact that the suspension of NP-RA was colloidal, the relatively large hydrodynamic size is related to the exceedingly good solvation of the RA chains by toluene. The increase in the hydrodynamic size from 30 nm to 45 nm during the polymerization is clearly related to the copolymerization of the NP-MMA and MMA. The relatively large hydrodynamic size and the increased skewness of the hydrodynamic size-distribution function of the NP-PMMA-3 suggest that some NPs were linked together; however, the number of the individual NPs linked together is quite low. This indicates that branched structures with NPs acting as the branching points were most likely formed instead of a fully cross-linked gel. The fact that the hydrodynamic size of the NP-PMMA nanoparticles was still large in the diluted sample was a further proof that the polymer chains were strongly attached, not only adsorbed onto the surface of the nanoparticles.

**Figure 4:**
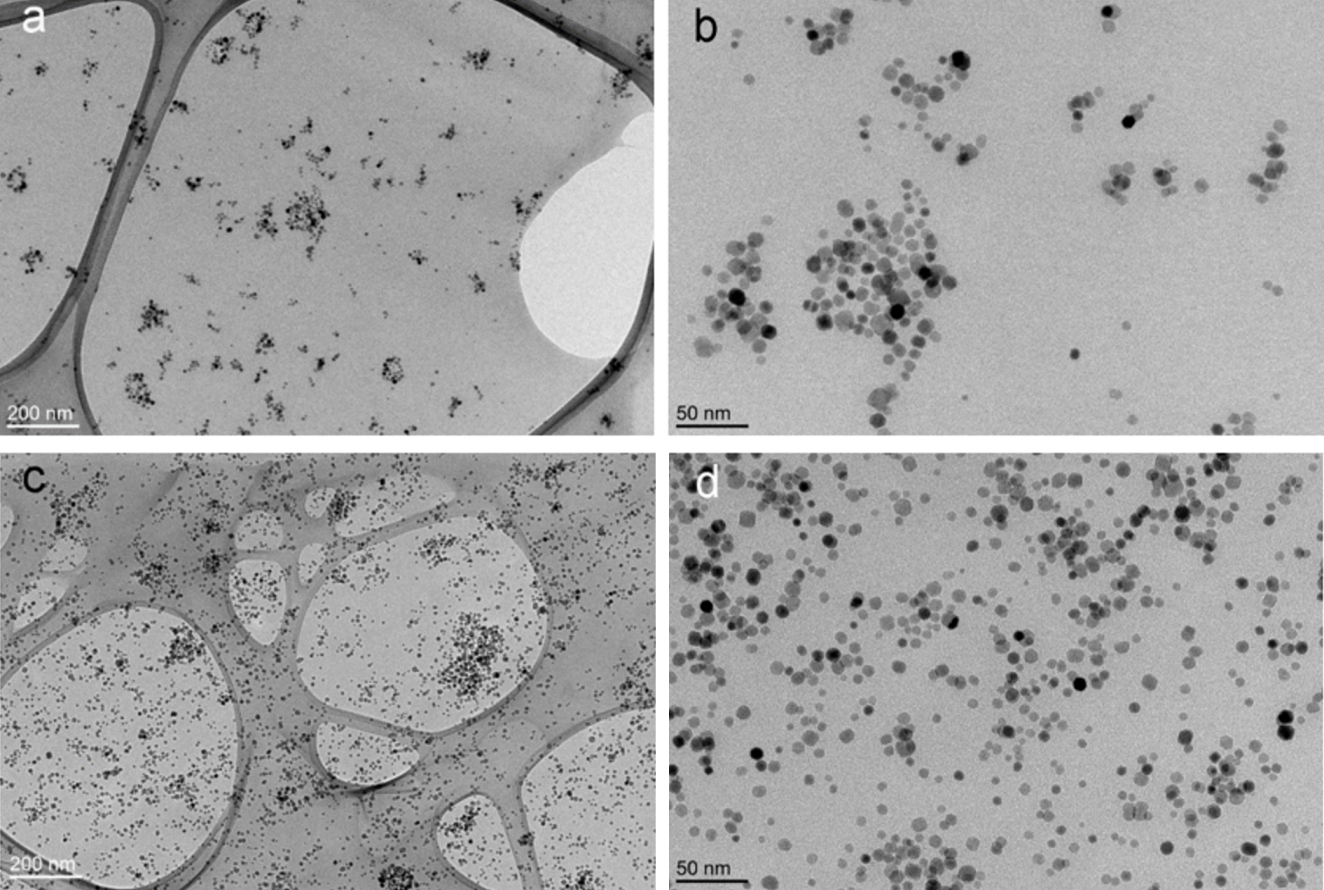
Cross-sectional TEM images of the nanocomposite sample NC-1 at lower (a) and higher (b) magnification and NC-3 at lower (c) and higher (d) magnification.

The weight fraction of the incorporated magnetic nanoparticles was determined from the TGA curves (Supporting Information File 1, Figure S7) and was found to be 5.2 wt %, 24.4 wt %, and 52.0 wt % for the nanocomposite samples NC-1, NC-2, and NC-3, respectively. The amount of incorporated nanoparticles was considerably larger than that calculated from the mass balance (assuming 100% conversion of the monomer). The larger amount of incorporated nanoparticles is related to the lower yield of precipitation of PMMA, decreasing the amount of isolated PMMA. PMMA is not soluble in pure methanol, however, in our case we precipitated nanocomposites by adding methanol to the suspension in toluene. In the precipitation mixture of toluene and methanol, a small amount of the PMMA, especially low molecular mass fractions, are soluble and were removed during washing of the nanocomposites. The dispersion of NP-PMMA nanoparticles in the nanocomposites is uniform, regardless of their content (Figure 4). In the NC-1 sample, the nanoparticles were predominantly dispersed as clusters; however, single nanoparticles were clearly visible (Figure 4a). The dispersion of the nanoparticles in the PMMA matrix was even more uniform as the amount of nanoparticles was increased to 52 wt % in the case of the NC-3 sample (Figure 4b).

The glass-transition temperature (*T*_g_) was found to be 105 °C for samples NC1 and NC2, and 124 °C for sample NC3. The *T*_g_ for the NC1 and NC2 samples was practically the same as that for pure PMMA [15]. On the other hand, the *T*_g_ of the NC3 sample is significantly higher, which points to a more rigid structure since the PMMA chains connect several neighboring nanoparticles.

Leaching of the Fe cations from the nanocomposites under physiological conditions can limit their use. We tested the dissolution of NPs from the nanocomposite under much harsher conditions (see Supporting Information File 1). For comparison, we performed experiments on samples NP-RA, NP-RA-PMMA and NC-3 in a solution of citric acid. Under sufficiently acidic conditions (pH < 6) the citric acid boosts the dissolution of iron oxide due to the chelate effect [32]. The results are presented as the wt % of dissolved Fe relative to the total Fe in the iron oxide nanoparticles. It was found that 58 wt % of Fe dissolved in the case of NP-RA, 33 wt % in the case of NP-RA-PMMA, and only 0.15 wt % in the case of NC-3. Despite the hydrophobic character of the NP-RA sample, the RA was insufficient to prevent the dissolution of the NPs. The dissolution was not much improved in the case of NP-RA-PMMA, where PMMA was not bonded to the surface of the agglomerated nanoparticles. Only negligible Fe dissolution was observed in the case of the NC-3 sample with the highest amount of incorporated nanoparticles. The exceedingly low dissolution is a consequence of the surface-bonded PMMA chains of less segment mobility that effectively prevented the access of the H^+^ and citrate anion to the surface of the NP-PMMA nanoparticles.

**Figure 5:**
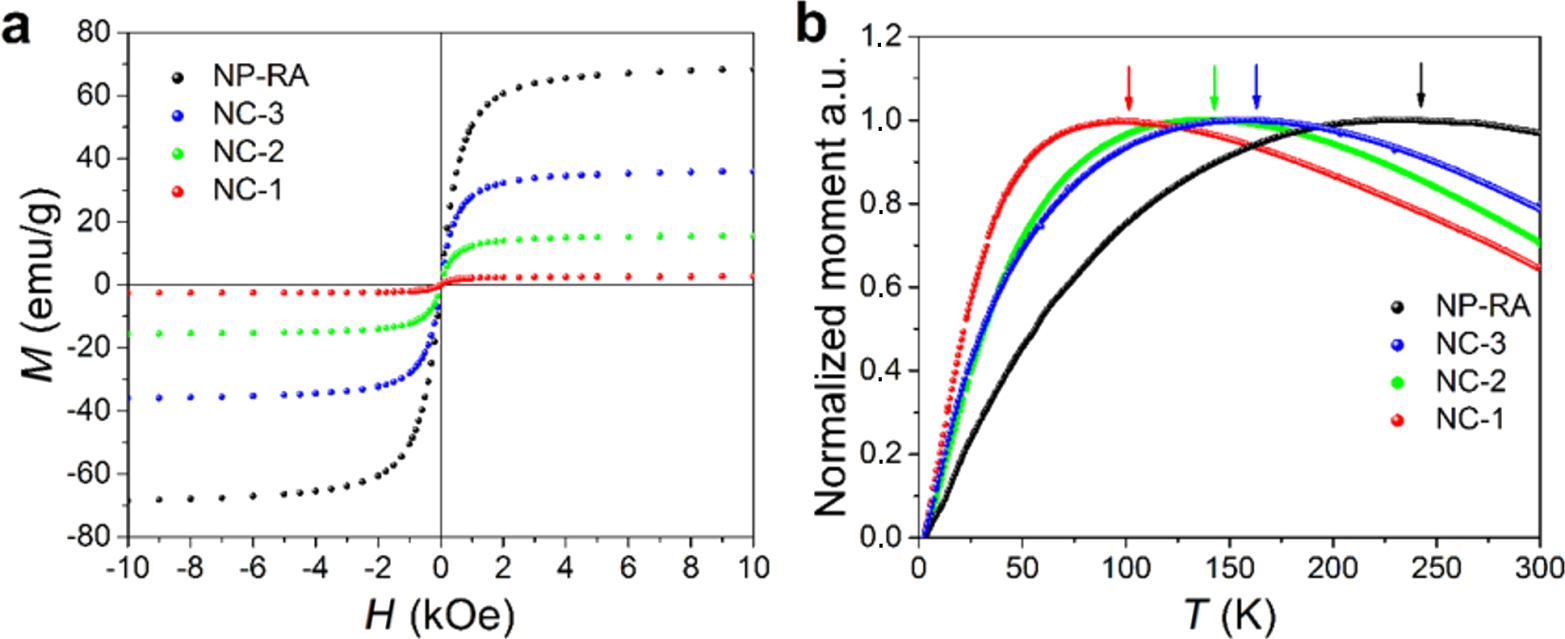
(a) Room-temperature magnetization curves of the NP-RA nanoparticles and nanocomposites. (b) Temperature dependence of the magnetic moment under zero-field cooling conditions for the NP-RA nanoparticles and nanocomposites.

Superparamagnetic behavior of the NP-RA nanoparticles and nanocomposites is evident from the room-temperature magnetization curves (Figure 5a). The saturation magnetization curves were consistent with the size of the nanoparticles and their amount incorporated into the nanocomposites [32–33]. The superparamagnetic nature was evident from the ZFC measurements (Figure 5b). For all of the samples, the maximum of the ZFC curve is below room temperature. The temperature of the maximum *T*_p_ is related to the characteristic blocking temperature, *T*_B_. For a system of magnetically non-interacting monodispersed nanoparticles, the *T*_p_ and *T*_B_ values are the same and correspond to the transition from the blocked to the superparamagnetic state [34].

The blocking temperature is mainly related to the average size of the nanoparticles. However, the dipolar interactions modify the collective magnetic behavior and their strength is increased as the interparticle separation decreases. Simply stated, the measured *T*_p_ increased as the interparticle separation becomes smaller for the particles belonging to the same size distribution. The NP-RA nanoparticles were only coated with the collapsed chains of ricinoleic acid and were strongly interacting. Their measured *T*_p_ is high. The *T*_p_ of the nanocomposites was lower than the *T*_p_ of the NP-RA and smoothly decreased as the amount of incorporated nanoparticles was reduced (Figure 5b). Two conclusions can be drawn from the observed behavior: firstly, that the NPs in the nanocomposites were not agglomerated and, secondly, that the interparticle separation increased as their amount decreased, suggesting that their dispersion is uniform. If the nanoparticles were agglomerated the *T*_p_ of the nanocomposites would not change with the amount of NPs and would be close to the *T*_p_ of the NP-RA.

**Figure 6:**
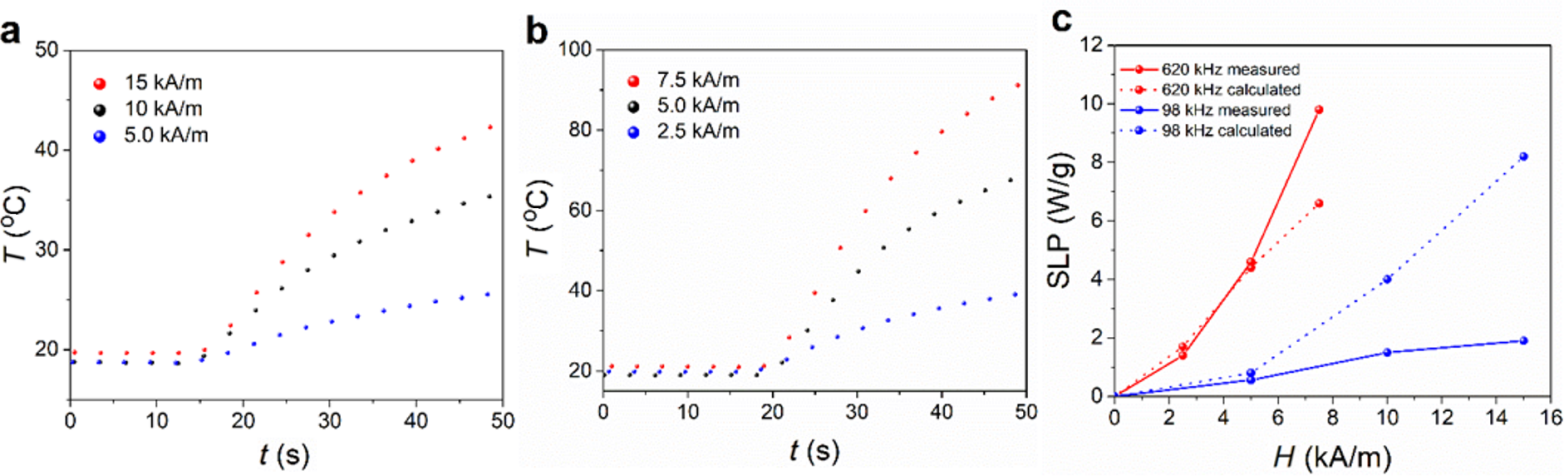
Heating curves of the NC3 sample measured at different AC field amplitudes at a frequency of (a) 98 kHz and (b) 620 kHz. (c) Calculated and measured specific power loss (SLP) as a function of the AC field amplitude measured at two frequencies.

The heating ability of the NC3 sample in an AC field was evaluated at two frequencies and different AC field amplitudes (Figure 6a and Figure 6b). The heating of the magnetic nanoparticles in the AC field is a consequence of the magnetic moment relaxation of the magnetic nanoparticles [35–36]. The heating and the characteristic value related to the heating ability, called the specific power loss (SLP), will be the greatest when the frequency of the AC field corresponds closely to the relaxation time of the nanoparticles. More specifically, in our case, this corresponds to the Néel relaxation time because the particles are fixed in the polymer and therefore Brownian relaxation is not possible. The important point is that even at low frequency and AC field amplitudes, the NC3 sample can produce a substantial amount of heat. At a frequency of 620 kHz and a relatively small AC amplitude of 5.0 kA/m (6 mT), the NC3 sample was rapidly heated to temperatures above 43 °C. This temperature is well beyond the temperature causing necrosis of cancer cells, which makes it an attractive material for cancer treatment using magnetic-field-induced hyperthermia [37].

As expected, the temperature measured at 620 kHz is much higher even at lower applied magnetic field fields than in the case of 98 kHz. Figure 6c shows the comparison of the expected SLP, calculated on the basis of the method shown by Carrey, taking into consideration the size distribution of the iron-oxide nanoparticles [36]. At the frequency of 620 kHz, the calculated and measured values are very similar. For 98 kHz, the measured SLP values are, especially at higher magnetic field strengths, clearly lower than the calculated ones. The reasons for this are the magnetic dipolar interactions at this high particle concentration and the applied field strengths, which lead to a longer relaxation time, and therefore, to a lower SLP value at the given frequency [38–39].

## Conclusion

We have demonstrated a simple approach to the preparation of nanocomposites with a high, uniform distribution of magnetic nanoparticles. The key is the copolymerization of MMA with iron-oxide nanoparticles bearing a methacrylate-based monomer. The PMMA chains on the surface of the nanoparticles provide excellent colloidal stability and prevent the agglomeration of the nanoparticles during the preparation of the nanocomposites. The PMMA chains are strongly attached to the surfaces of the nanoparticles, as indicated by the inhibited Fe ion dissolution under highly acidic conditions. The nanocomposites exhibit superparamagnetic behavior and large saturation magnetization values, in accordance with their content. The nanocomposite with the highest loading of magnetic nanoparticles was found to generate a substantial amount of heat when exposed to a relatively low amplitude of the AC magnetic field, and thus has the potential for applications in cancer treatment based on magnetically induced hyperthermia.

## Supporting Information

Additional description of materials used and the synthesis and characterization of nanocomposite materials. Additional FTIR and NMR spectra, TGA curves, a correlation function of the suspensions and TEM images showing the size distribution function of NP-RA are given.

File 1 Additional experimental information.
