# Supplementary material for: Nanocomposites comprised of homogeneously dispersed magnetic iron-oxide nanoparticles and poly(methyl methacrylate)
Source: Beilstein J Nanotechnol. 2018 Jun 1;9:1613–22. doi: 10.3762/bjnano.9.153 (PMC6009273; doi:10.3762/bjnano.9.153)
Supplement: File 1 — Additional experimental information. [file Beilstein_J_Nanotechnol-09-1613-s001.pdf]

Supporting Information

for

# Nanocomposites comprised of homogeneously dispersed magnetic iron-oxide nanoparticles and poly(methyl methacrylate)

Sašo Gyergyek[1,2*], David Pahovnik[3], Ema Žagar[3], Alenka Mertelj[4], Rok Kostanjšek[5], Miloš Beković[6], Marko Jagodič[7], Heinrich Hofmann[8], and Darko Makovec[1]

Address: [1]Department for Materials Synthesis, Jožef Stefan Institute, Jamova 39, 1000 Ljubljana, Slovenia, [2]Faculty of Chemistry and Chemical Engineering, University of Maribor, Smetanova 17, 2000 Maribor, Slovenia, [3]Department of Polymer Chemistry and Technology, National Institute of Chemistry, Hajdrihova 19, 1000 Ljubljana, Slovenia, [4]Complex Matter, Jožef Stefan Institute, Jamova 39, 1000 Ljubljana, Slovenia, [5]Department of Biology, Biotechnical Faculty, University of Ljubljana, Večna pot 111, 1000 Ljubljana, Slovenia, [6]Institute of Electrical Power Engineering, Faculty of Electrical Engineering and Computer Science, University of Maribor, Smetanova 17, 2000 Maribor, Slovenia, [7]Institute of Mathematics, Physics and Mechanics, Jadranska 19, 1000 Ljubljana, Slovenia, and [8]Laboratory for Powder Technology, Ecole Polytechniquie Fédérale de Lausane, Station 12, 1015 Lausane, Switzerland

Email: Sašo Gyergyek - saso.gyergyek@ijs.si

* Corresponding author

## Additional experimental information

**Materials**

Iron(III) sulphate hydrate ($Fe_2(SO_4)_3 \cdot xH_2O$, reagent grade), iron(II) sulphate ($FeSO_4 \cdot 6H_2O$, 98%), methyl methacrylate (MMA, 99%, stabilized), pyridine (99+%) were purchased from Alfa Aesar. Ricinoleic acid (technical, <80%) was purchased from TCI ($^1$H NMR and FTIR spectra are given in Supplementary Information). Ammonia solution (25% for analysis), toluene (ACS reagent), methanol (Chromasolv grade), 2,2′-azobis(2-methylpropionitrile) (AIBN, 98%), methacrylic anhydride (technical, 94%, stabilized) and citric acid (anhydrous, ACS reagent) were purchased from Sigma-Aldrich. Nitric acid (65%) and acetone (RPE) were purchased from Carlo-Erba. KBr (for IR spectroscopy) was purchased from Merck. Aluminium oxide (neutral, Brockman I) was purchased from Acros Organics. Stabilizers from MMA and methacrylic anhydride were removed by passing the monomers over the aluminium oxide. The MMA was thoroughly bubbled with Ar and kept at room temperature in the dark under a slight Ar over-pressure. The methacrylic anhydride was stored in a fridge. Other chemicals were used as received.

**Characterization**

The particle size, crystallinity and dispersion of the nanocomposites was characterized by transmission electron microscopy (TEM). The thermionic electron-source TEM (Jeol JEM-2100) was operated at 200 kV. For the TEM investigation of the nanoparticles the suspension of the nanoparticles NP-MMA was deposited on a copper-grid-supported, perforated, transparent carbon film. For the TEM investigation of the nanocomposites, the powders were first hot pressed into 5 mm x 5 mm x 8 mm blocks, then mounted in a holder and the area was decreased to approx. 2 mm x 2 mm using a blade. 50-nm-thick sections were prepared on an Ultracut S ultramicrotome (Reichert) and transferred to a copper-grid-supported, transparent

carbon film. $^1$H NMR spectra were recorded in $CDCl_3$ on a 300-MHz Agilent Technologies DD2 NMR spectrometer in the pulse Fourier Transform mode with both a relaxation delay and an acquisition time of 5 s. Tetramethylsilane (TMS, $\delta = 0$) was used as an internal chemical-shift standard. Diffuse reflectance infrared Fourier transform spectra (DRIFT, Perkin Elmer Spectrum 400 equipped with DRIFT accessory) were recorded in KBr. The spectra were averaged over 64 scans recorded with a resolution of 1 $cm^{-1}$. The FTIR spectrum of technical ricinoleic acid was recorded with the ATR technique (Perkin Elmer Spectrum 400 equipped with ATR accessory). The spectrum was averaged over 16 scans, recorded with a resolution of 4 $cm^{-1}$. The weight fraction of nanoparticles in the nanocomposites was determined with a Mettler Toledo thermogravimetric analysis (TGA) instrument equipped with STARe 9.3 software (Mettler Toledo, OH, USA). Samples were heated from room temperature to 600 °C in $N_2$ and from 600 °C to 1000 °C in $O_2$ at a heating rate of 10 °C/min. The glass-transition temperature of the nanocomposites was determined on a Mettler Toledo DSC1 at a heating rate of 20 °C/min from 0 °C to 150 °C, followed by cooling at 200 °C/min to 0 °C, and a second heating with a rate of 20 °C/min back to 150 °C. Glass-transition temperatures were obtained from the second heating. Dynamic light scattering (DLS) was used to measure a distribution of nanoparticles hydrodynamic sizes. A standard photon correlation setup with a He-Ne laser and an ALV-6010/160 correlator was used to obtain the autocorrelation function of the scattered light intensity. The scattered light was collected with a monomode optical fibre, with a built in GRIN lens. The light was detected by an ALV APD based “pseudo” cross correlation detector. The measurements were performed at a scattering angle of 90°, except for sPMMA in which case the scattering angle was

30° (Scheme S1). The radius distribution functions were calculated using the CONTIN analysis, which is part of the ALV-Correlator Software V3.0.

The correlation functions measured in the diluted (below 1 vol %) suspensions of the original magnetic particles (NP-RA) and the isolated nanoparticles NP-PMMA-3 in toluene are shown in Figure S9. The correlation function measured in the NP-RA suspension is noisier due to weaker scattering from magnetic particles. The main decay of the correlation function in the NP-RA case is clearly faster than that in NP-PMMA-3 indicating smaller particles size. In the NP-PMMA-3 case an additional small faster relaxation can be observed. Using CONTIN analysis the distribution of the relaxation rates was obtained, which was transformed to the number weighted distribution of the particles diameters assuming spherical particles (again an option built in the ALV-Correlator Software V3.0).

The room-temperature magnetization curves of the nanoparticles were measured with a Lake Shore 7307 vibrating-sample magnetometer (VSM). The temperature dependency of the magnetic susceptibility under zero-field-cooling (ZFC) conditions was measured with a Quantum Design MPMS superconducting quantum interference device (SQUID). The SLP of the nanoparticles in powder form was measured using a custom-built device. The measuring system consists of a power supply coil, appropriate capacitors for the LC-resonant circuit, a function generator for selection of frequencies, and a power amplifier. The coil is a double-layered, made of a copper tube and water-cooled. Its axial length of 20 cm provides a homogeneous magnetic field for the measurement sample in the center of the coil. Sample in a plastic holder is inserted in a heat chamber connected to a heat-bath. The automated measurement includes measurement of temperature with a fiber-optic temperature

sensor (FISO) insensitive to high-frequency magnetic fields and measurement of the magnetic field strength using a measuring coil.

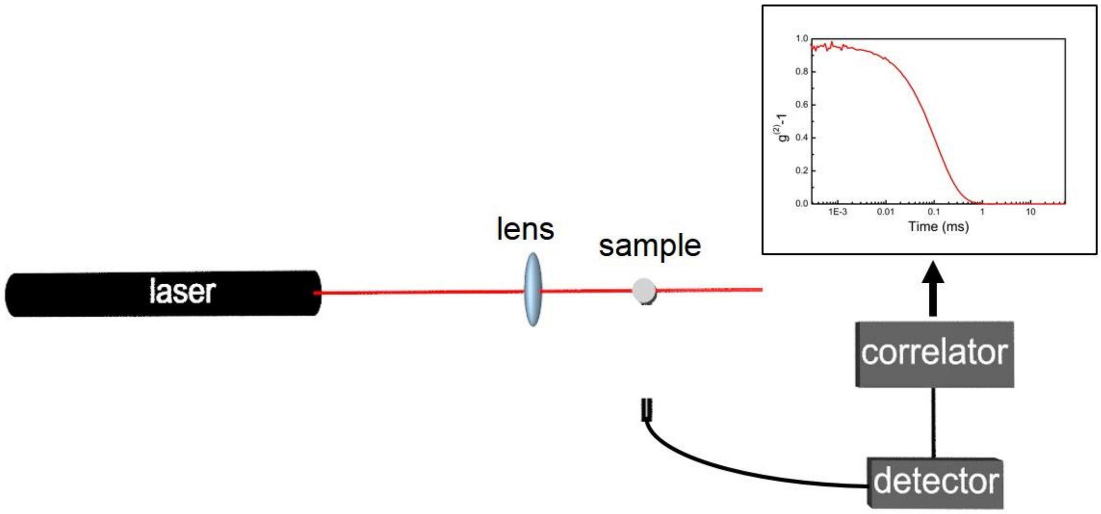


**Scheme S1:** Schematic representation of the DLS setup.

**Synthesis**

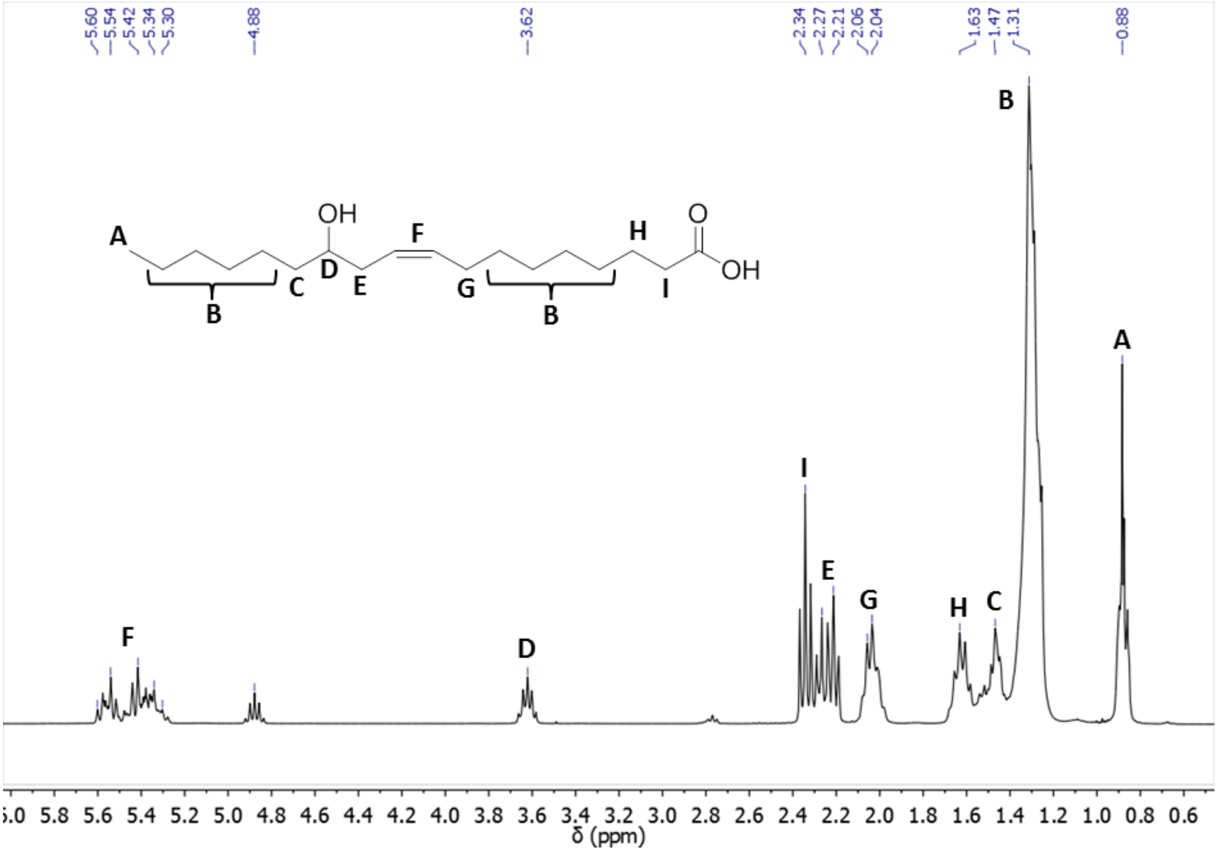


**Figure S1:** $^{1}H$ NMR spectrum of technical grade ricinoleic acid with peak assignments.

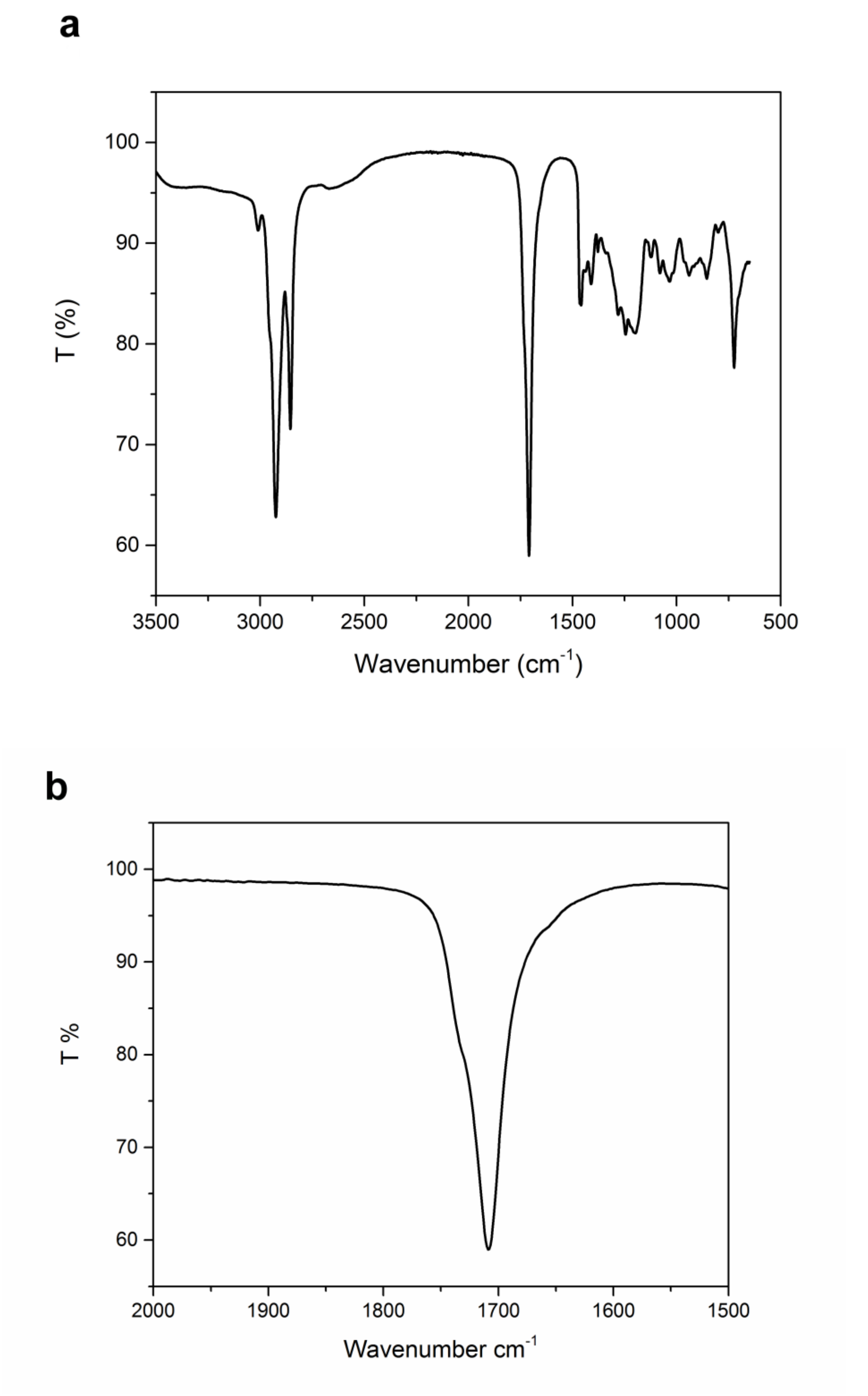


**Figure S2:** FTIR spectrum of technical grade ricinoleic acid (a) and enlarged area of the same spectrum (b).

**Synthesis of RA-MMA**

5 g of ricinoleic acid (16.7 mmol) was dissolved in 52 mL of toluene in a 100 mL round-bottom flask. To the above solution, 2.705 mL (18.2 mmol) of methacrylic anhydride and 1.5 mL (18.6 mmol) of pyridine were added. The flask was sealed with septum and heated in an oil bath at 80 °C for 48 h. The organic phase was washed with water and separated from the aqueous layer. Toluene as removed under vacuum. The product (RA-MMA) obtained was a yellowish viscous liquid.

**$^{1}$H NMR**

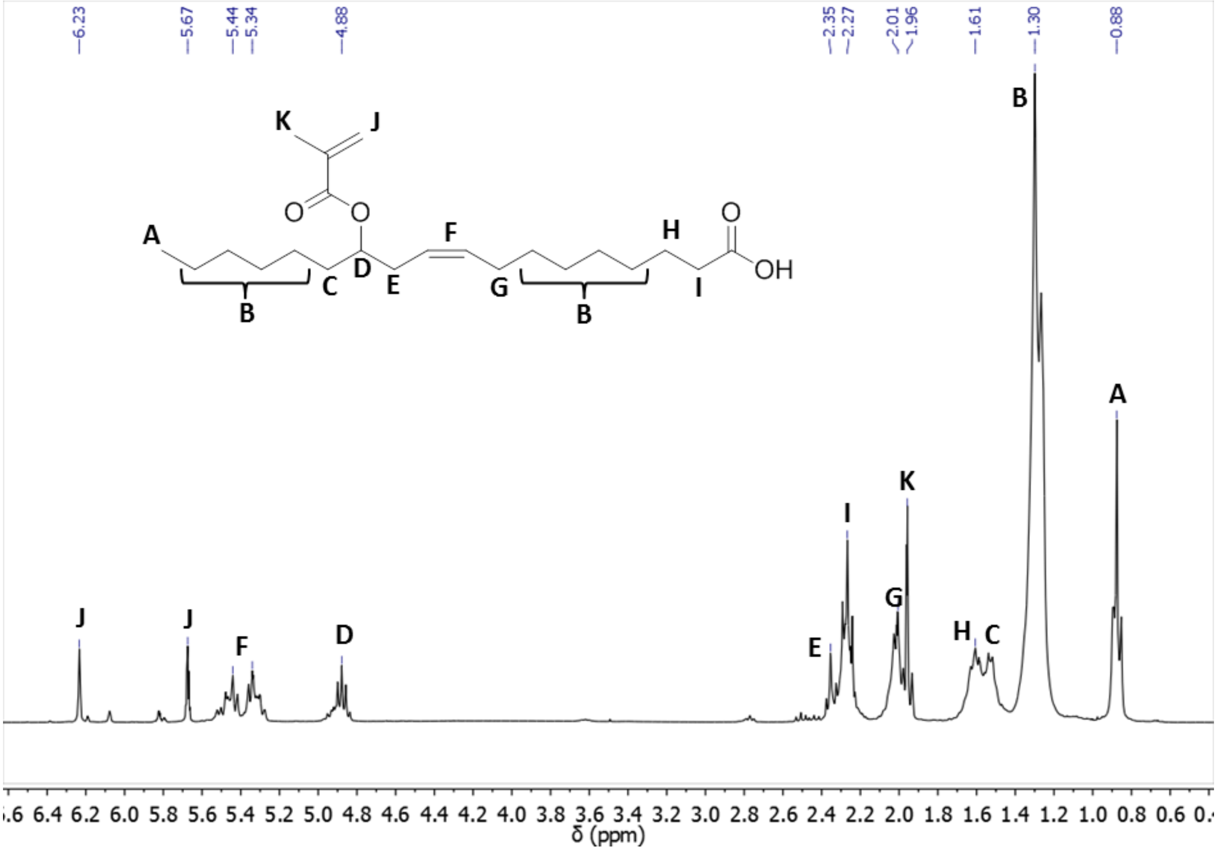


**Figure S3:** $^{1}$H NMR spectrum of RA-MMA with peak assignments.

**Polymerization of RA-MMA**

A 10 mL round-bottom flask was charged with 1.2 g of RA-MMA, 0.017 g of AIBN and 3.8 mL of toluene. The solution was bubbled with Ar gas. After 30 minutes the bubbling was stopped, 1.4 mL of MMA was added and the solution was heated at 80 °C for 4 h. The product was dried for 24 h at 60 °C and 2 h at 105 °C. The product (RA-PMMA) obtained was a yellowish soft solid.

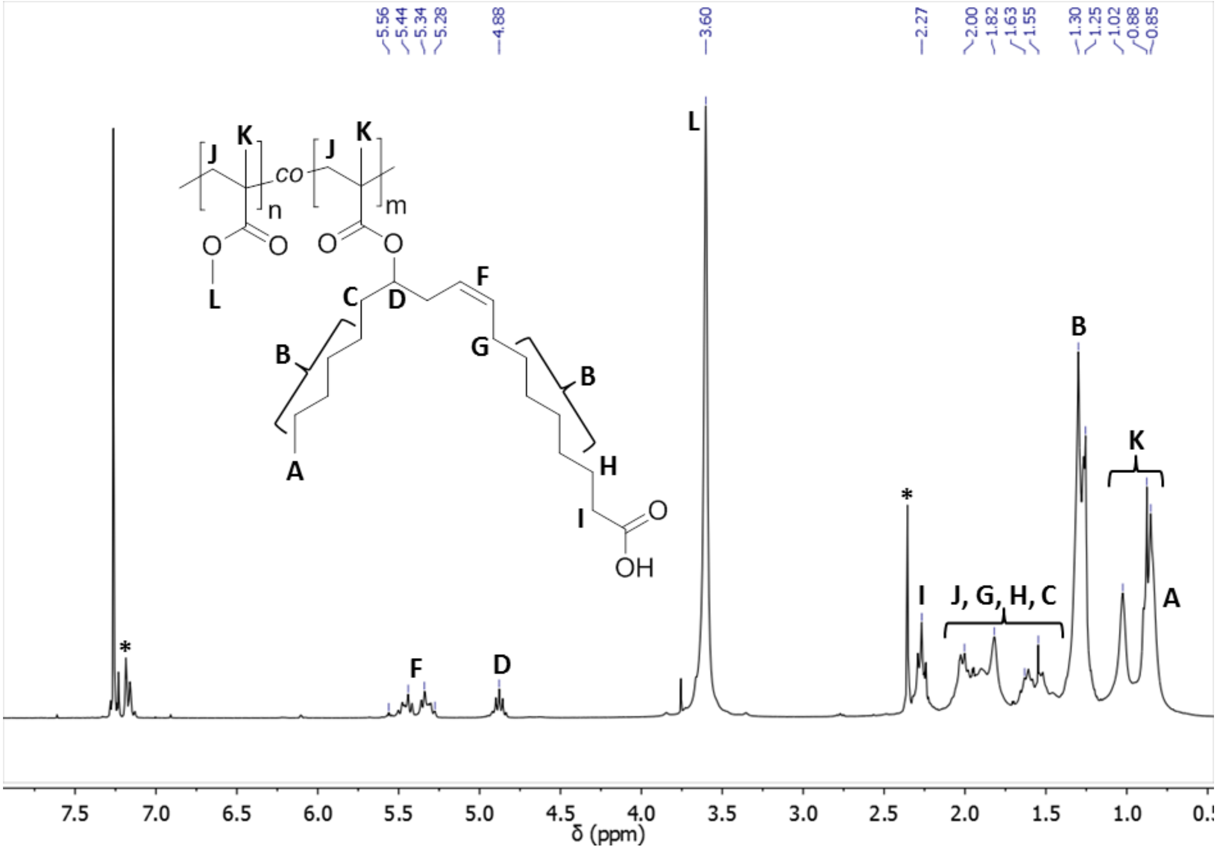


**Figure S4:** $^1$H NMR spectrum of RA-PMMA with peak assignments (* denotes residual toluene).

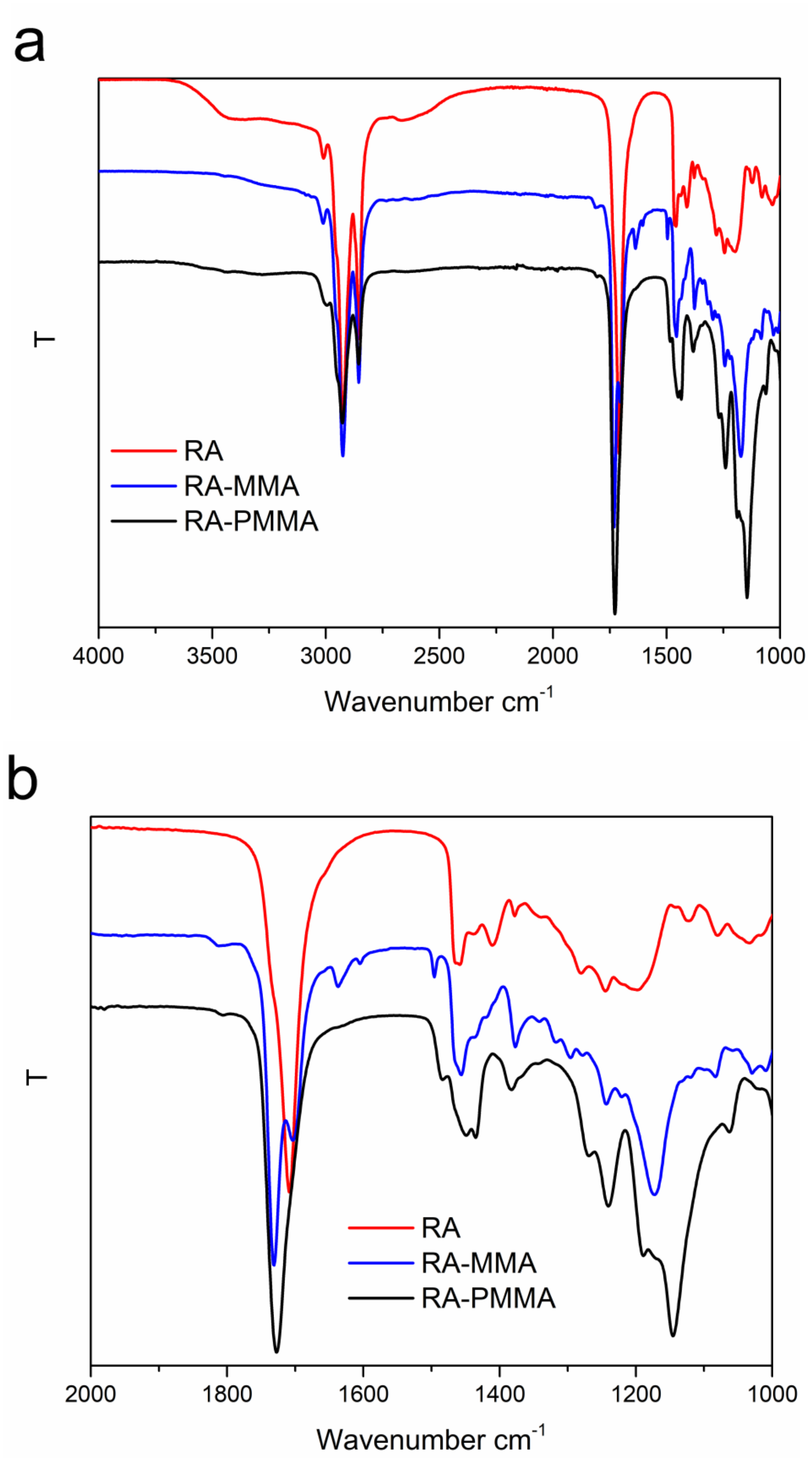


**Figure S5:** FTIR spectrum of technical grade ricinoleic acid RA, RA-MMA and RA-PMMA (a) and enlarged area of the same spectrum (b).

## NP-RA-PMMA

0.36 g of the suspension of the nanoparticles NP-RA was mixed with the 2.8 g of the solution of the sPMMA. Immediately after mixing half of the suspension was poured into a large excess of methanol to precipitate the nanocomposite NP-RA-PMMA. The nanocomposite was washed several times with methanol, oven dried at 60 °C and crushed in an agate mortar. The nanocomposite contained 13.9 wt.% of the nanoparticles NP.

## Dissolution of the nanoparticles

10 mL of aqueous solution of the citric acid at pH 1 was added in three 20 mL glass vials containing 50 mg of the nanoparticles NP-MMA, nanocomposites M-NP-RA-PMMA and NC-3, each. Vials were shaken for 12 days at room temperature. Undissolved magnetic material was separated using a permanent magnet, the decanted solution was filtered and analysed. The iron content in the solutions was determined using inductively coupled plasma optical emission spectroscopy (ICP-OES).

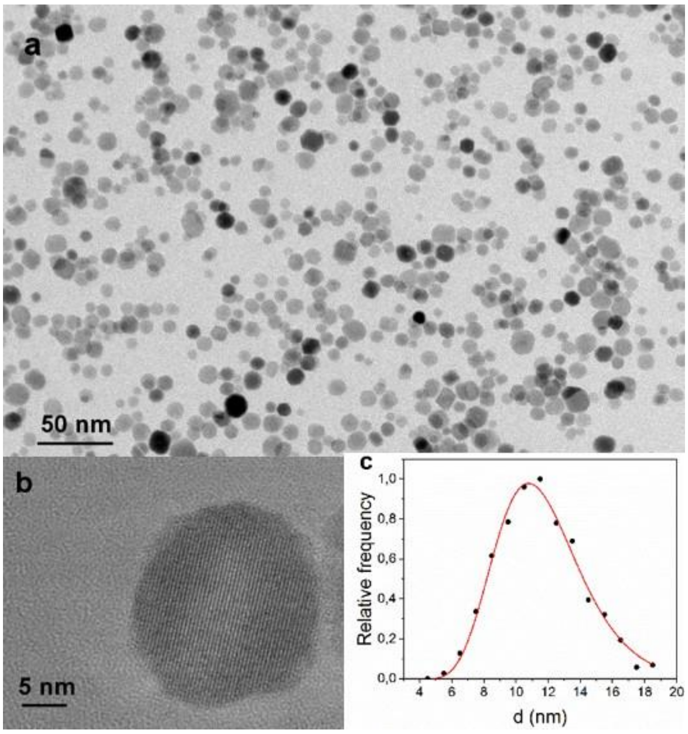


**Figure S6:** TEM image and empirical size distribution of nanoparticles NP-RA.

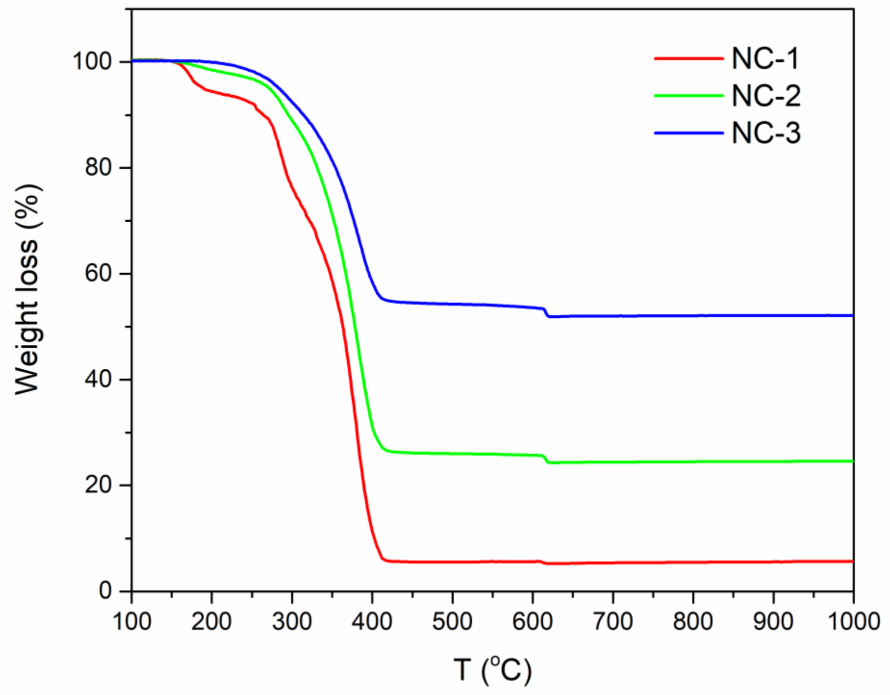


**Figure S7:** TGA curves of the nanocomposites.

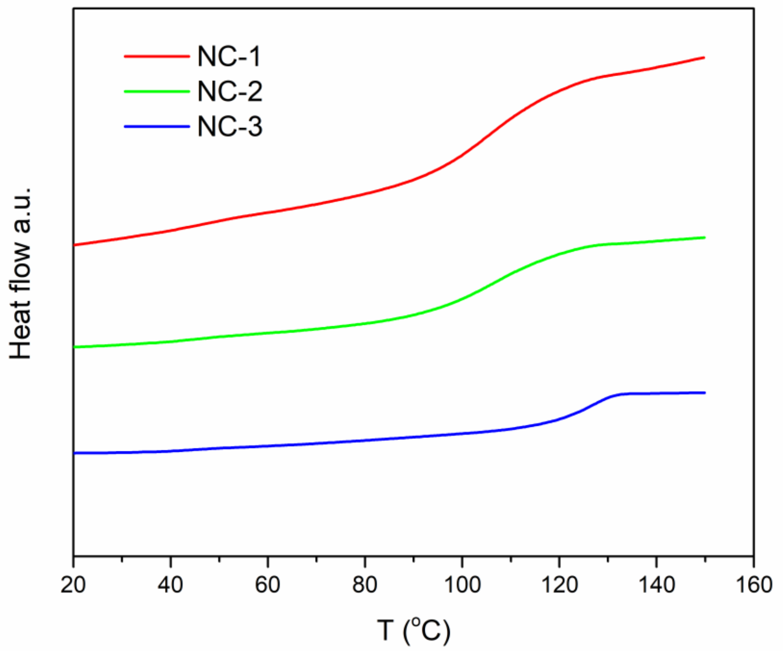


**Figure S8:** DCS curves of the nanocomposites.

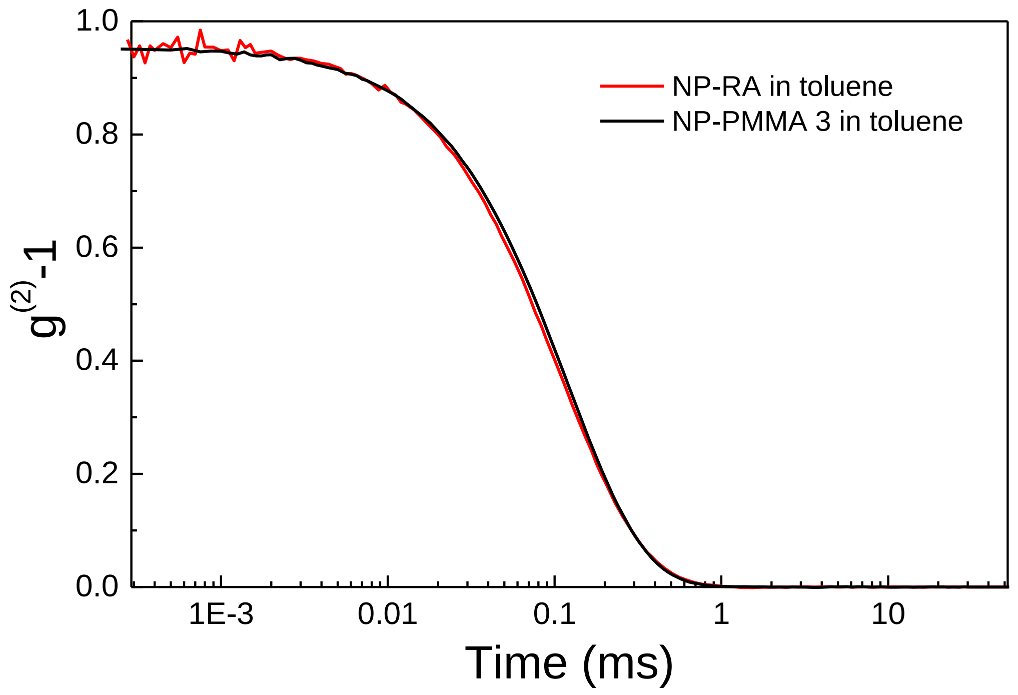


**Figure S9:** Correlation functions of the suspensions NP-RA and NP-PMMA-3.
